# Hepatic fascioliasis: a rare case of “fake” biliary gallstones

**DOI:** 10.1055/a-2254-0187

**Published:** 2024-02-22

**Authors:** Michele Amata, Giovanni Boncori, Daniela Scimeca, Filippo Mocciaro, Ambra Bonaccorso, Claudia Colomba, Roberto Di Mitri

**Affiliations:** 1 Gastroenterology and Endoscopy Unit, ARNAS Civico Di Cristina Benfratelli Hospital, Palermo, Italy; 2 Department of Health Promotion, Maternal and Infant Care, Internal Medicine and Medical Specialties, University of Palermo, Palermo, Italy; 3 Division of Pediatric Infectious Diseases, “G. Di Cristina” Hospital, ARNAS Civico Di Cristina Benfratelli Hospital, Palermo, Italy

A 52-year-old woman who had previously traveled to a developing country was admitted to our emergency department with fever, colicky abdominal pain, and pruritus. Laboratory tests showed hypereosinophilia (1432 eosinophils/µL, limit value <500) and direct hyperbilirubinemia (2.5 mg/dL, reference range 0–1.3 mg/dL). Magnetic resonance cholangiopancreatography revealed endoluminal filling defects into a dilated common bile duct (CBD) and in the gallbladder ( Fig. 1 ). Endoscopic retrograde cholangiopancreatography (ERCP) was then planned to treat choledocholithiasis.

**Fig. 1:**
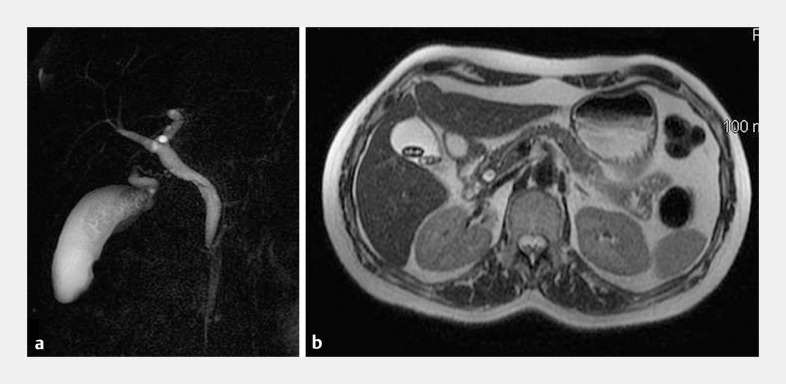
Imaging studies showing a small defect inside the common bile duct, with two suspected stones inside the gallbladder with flat shape and atypical morphology. **a** Magnetic resonance cholangiopancreatography. **b** Axial fast imaging employing steady-state acquisition magnetic resonance imaging.

Using the double-wire technique, deep biliary cannulation was performed and fluoroscopic cholangiogram confirmed an irregular filling defect in the distal CBD. Surprisingly, after biliary sphincterotomy, a live and mobile flat-shaped worm was extracted from the CBD by a balloon catheter and retrieved using a biopsy forceps ( Fig. 2 , Video 1 ). Macroscopically, the parasitic trematode presented with a whitish, flat, and elongated morphology, 25 × 10 mm in size, compatible with *Fasciola hepatica* ; it was also confirmed at the microscopic analysis by the evidence of a pathognomonic spiny tegument.

**Fig. 2:**
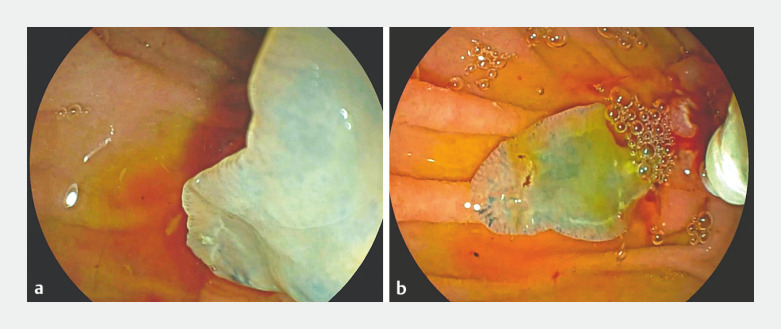
*Fasciola hepatica* extracted from the biliary tree. At the macroscopic evaluation, the trematode presented as a flat leaf-shaped hermaphrodite fluke, gray in color. The adult worm may live in the biliary tract of the definitive host for many years (5 years in sheep and 10 years in humans).


Endoscopic extraction of *Fasciola hepatica* from the biliary tree.
Video 1

Following a course of antiparasitic drug (triclabendazole 10 mg/kg oral solution, twice a day for 2 days), laparoscopic cholecystectomy was performed and confirmed only the presence of two gallstones. The patient was asymptomatic at the 4-month follow-up.

*F. hepatica* is a leaf-shaped trematode that usually attacks cattle and sheep ( Fig. 3 ), and is frequently found in endemic and developing countries. Humans may become accidental hosts through drinking water or ingesting raw green vegetables contaminated with encysted metacercariae. The parasite larva penetrates the intestinal wall and Glisson’s capsule, colonizing the biliary tree 1 . Living or dead *F. hepatica* may occlude the bile ducts, causing obstruction and sometimes cholangitis. ERCP is fundamental for diagnosis and the mechanical removal of *F. hepatica* from the CBD 2 , and triclabendazole allows complete eradication as it is active against both immature and adult parasites.

**Fig. 3:**
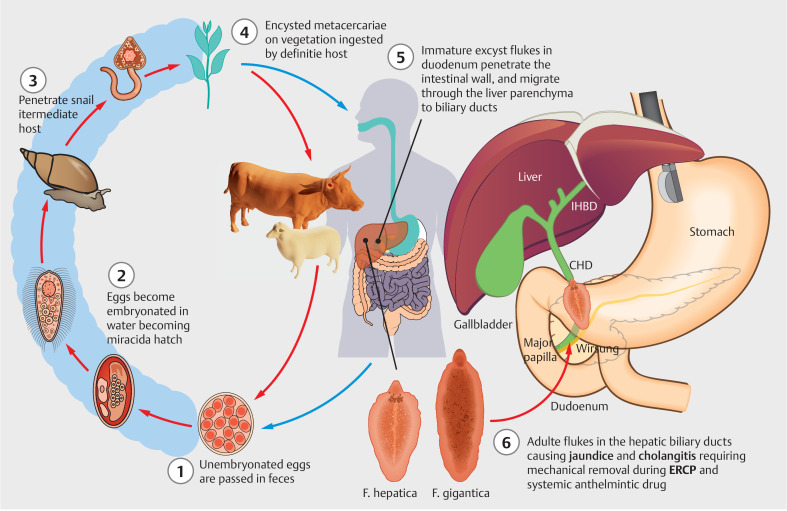
Life cycle of *Fasciola hepatica* (graphical illustration by Michele Amata, MD). CHD, common hepatic duct; ERCP, endoscopic retrograde cholangiopancreatography; IHBD, intrahepatic bile duct.

Endoscopy_UCTN_Code_CCL_1AC_2AG

## Notes

Endoscopy E-Videos https://eref.thieme.de/e-videos
*E-Videos* is an open access online section of the journal *Endoscopy* , reporting on interesting cases and new techniques in gastroenterological endoscopy. All papers include a high-quality video and are published with a Creative Commons CC-BY license. Endoscopy E-Videos qualify for HINARI discounts and waivers and eligibility is automatically checked during the submission process. We grant 100% waivers to articles whose corresponding authors are based in Group A countries and 50% waivers to those who are based in Group B countries as classified by Research4Life (see: https://www.research4life.org/access/eligibility/ ). This section has its own submission website at https://mc.manuscriptcentral.com/e-videos .
